# CD80-Mediated T-Cell Suppression by Cancer Stem-like Cells in Head and Neck Squamous Cell Carcinoma

**DOI:** 10.3390/cells15030266

**Published:** 2026-01-30

**Authors:** Mian Xiao, Lin Qiu, Qian Gao, Ruifeng Li, Jing Wang, Yanrui Feng, Xuefen Li, Xiyuan Ge

**Affiliations:** 1 Central Laboratory, Peking University School and Hospital of Stomatology, Beijing 100081, China; 2 National Clinical Research Center for Oral Diseases, Beijing 100081, China; 3 National Engineering Laboratory for Digital and Material Technology of Stomatology, Beijing 100081, China; 4 Beijing Key Laboratory of Digital Stomatology, Beijing 100081, China; 5 Department of Oral and Maxillofacial Surgery, Peking University School and Hospital of Stomatology, Beijing 100081, China

**Keywords:** head and neck squamous cell carcinoma, neoadjuvant chemoimmunotherapy, cancer stem-like cells, CD80, therapeutic resistance

## Abstract

Neoadjuvant chemoimmunotherapy has emerged as a promising treatment strategy for head and neck squamous cell carcinoma (HNSCC). There is an urgent need to improve patient responses to this approach. In this study, we aim to elucidate the mechanisms underlying poor response to neoadjuvant chemoimmunotherapy and to identify strategies to enhance therapeutic efficacy in HNSCC. We identified a cancer stem-like cell (CSC) population enriched in patients with partial response (PR) to neoadjuvant chemoimmunotherapy, characterized by high CD80 expression. CD80 was likewise highly expressed in ALDH^high^CD44^+^ and BMI1^+^ populations. Functionally, CD80 knockdown attenuated tumor-sphere-forming capacity and reduced the migration and invasion of tumor cells, whereas CD80 overexpression potentiated these pro-tumorigenic activities. Moreover, CD80 inhibition activated signaling pathways of Th1 immune responses and IL-2 production. CD80 blockade enhanced T cell cytotoxicity. In preclinical HNSCC models, inhibition of CD80 significantly decreased tumor burden, accumulated CD8^+^ T cells, and increased the production of cytotoxic effector molecules. Our data demonstrated that CD80 modulated tumor-cell stemness and malignant phenotype while restraining antitumor T cell immunity. Targeting CD80 augments antitumor immunity and provides a compelling strategy to enhance treatment responses to neoadjuvant chemoimmunotherapy in HNSCC.

## 1. Introduction

Head and neck squamous cell carcinoma (HNSCC) is a highly aggressive tumor characterized by frequent therapeutic resistance [1,2,3]. Immunotherapy has emerged as a promising treatment modality for HNSCC by enhancing antitumor immunity and reshaping the tumor microenvironment [4,5]. While PD-1 monotherapy and dual immunotherapy remain suboptimal in HNSCC clinical trials [6,7], chemoimmunotherapy combining PD-1 blockade with chemotherapeutic agents has shown more encouraging outcomes. Neoadjuvant chemoimmunotherapy can alleviate the immunosuppression, remodel the tumor microenvironment, and activate antitumor immunity. A retrospective study of tislelizumab combined with chemotherapy in HNSCC reported that the objective response rate (ORR) was 66.7%. The combined therapy achieved better organ preservation and increased the chance of surgical treatment [8]. Consistently, a multicenter phase III trial of tislelizumab plus chemotherapy in nasopharyngeal cancer significantly promoted the patients’ progression-free survival (PFS) (9.6 months vs. 7.4 months) [9]. In a phase III trial of finotonlimab plus chemotherapy in recurrent or metastatic head and neck cancer, the experimental arm achieved a median overall survival of 14.1 months versus 10.5 months in the control arm [10]. Despite these advances, there is a pressing need to improve the response rate of HNSCC patients to neoadjuvant chemoimmunotherapy. Neoadjuvant chemoimmunotherapy may substantially reduce tumor burden, increase the chance of organ preservation, and significantly improve postoperative survival [11]. Thus, we need to elucidate the biological mechanisms underlying suboptimal responses to neoadjuvant chemoimmunotherapy.

Accumulating evidence implicates cancer stem-like cells (CSCs) as major determinants of tumor recurrence, metastasis, and resistance to therapies [12,13,14]. CSCs typically exhibit high-level expression of stemness-related genes [15]. Functionally, CSCs exhibit pronounced self-renewal ability and differentiation plasticity, and can drive tumor initiation even when present at very low frequencies [16,17]. Multiple studies across solid tumors have demonstrated that therapeutic targeting of CSCs can substantially impede tumor progression [18,19]. Thus, unraveling how CSCs sustain treatment resistance is therefore pivotal for identifying new therapeutic vulnerabilities and refining chemoimmunotherapy strategies.

In this study, we performed single-nucleus RNA sequencing (snRNA-seq) and integrative transcriptomic analyses on tumor specimens from HNSCC patients treated with neoadjuvant therapy. We identified a CSC subpopulation with elevated stemness signatures that was preferentially enriched in patients with poor responses. These CSCs exhibited robust CD80 overexpression. Thus, we hypothesize that CSCs contribute to immune resistance in HNSCC via CD80 upregulation. Consequently, targeting CD80 is a promising strategy to augment the clinical benefit of neoadjuvant therapy in HNSCC.

## 2. Materials and Methods

### 2.1. Human Samples

Formalin-fixed, paraffin-embedded (FFPE) tumor tissues from four patients with HNSCC treated at Peking University School and Hospital of Stomatology were collected for single-nucleus RNA sequencing (snRNA-seq). The tumor specimens were collected by biopsy before neoadjuvant therapy. Eligible patients were selected based on the following inclusion criteria: (1) confirmed diagnosis of HNSCC by two experienced pathologists; (2) clinical stage III–IV according to the eighth edition of the American Joint Committee on Cancer (AJCC) guideline. All four patients received two cycles of neoadjuvant tislelizumab in combination with cisplatin and paclitaxel before surgical resection. Neoadjuvant treatment efficacy was evaluated based on both clinical and pathological responses. Clinical response was assessed by changes in tumor volume measured on MRI scans obtained before and after neoadjuvant therapy and independently evaluated by two experienced surgeons. Pathological response was determined by the proportion of residual tumor cells in post-treatment specimens and assessed by two experienced pathologists. All four patients exhibited tumor volume reduction at the clinical level. Based on pathological response, patients were classified into partial response (PR) or complete response (CR) groups. Residual tumor cells were detected in PR tumors, whereas no tumor cells were observed in CR tumors. An overview of the case selection process is provided in Supplementary Figure S1. Patient characteristics are provided in Supplementary Table S1. Peripheral blood was obtained from healthy volunteers following informed consent. The study protocol was reviewed and approved by the Ethics Committee of Peking University School and Hospital of Stomatology (Beijing, China; approval no. PKUSSIRB-202169171/approval date: 15 November 2021).

### 2.2. Cell Lines

Human HaCaT keratinocytes and human HNSCC cell lines (HN6, CAL27, SCC9, SCC25) were obtained from the Central Laboratory, Peking University School and Hospital of Stomatology. Murine oral carcinoma cell lines MOC1 and MOC2 were acquired from Kerafast (Boston, MA, USA). HaCaT, HN6, and CAL27 cells were maintained in DMEM supplemented with 10% FBS and penicillin-streptomycin at 37 °C in a humidified 5% CO_2_ incubator. SCC9, SCC25, MOC1, and MOC2 cells were cultured in DMEM/F-12 supplemented with 10% FBS and penicillin–streptomycin under the same conditions.

### 2.3. SiRNA Knockdown and Overexpression

For transfection of CD80 siRNAs (RiboBio, Guangzhou, China), siRNAs were transfected with TransIT-X2^®^ Dynamic Delivery System (Mirus, Madison, WI, USA, Cat#MIR 6000) according to the manufacturer’s protocol. Three CD80 siRNA sequences have been shown in Supplemental Table S2. For transfection of CD80 plasmids, vector or CD80 plasmids (GeneChem, Shanghai, China) were transfected with TransIT-X2^®^ Dynamic Delivery System (Mirus, Madison, WI, USA, Cat#MIR 6000) according to the manufacturer’s protocol. The knockdown or overexpression of CD80 was confirmed by RT-qPCR.

### 2.4. Single-Nucleus RNA-Seq

FFPE samples were collected, and single-nucleus isolation and library preparation were performed at M20 Genomics (Hangzhou, China). FFPE tissue sections were washed with 1 mL xylene at room temperature to remove paraffin, followed by rehydration through a graded ethanol series. Samples were washed twice with pre-chilled wash buffer and homogenized on ice using a Dounce homogenizer (Bellco Glass, Inc., Vineland, NJ, USA, Cat. #50-194-5204) in pre-chilled lysis buffer (1 × PBS, 0.1% Triton X-100, 1 U/μL RNase inhibitor). After homogenization, the Dounce homogenizer was rinsed with 1 mL lysis buffer, and proteinase K (100 μL, 10 mg/mL; Sangon Biotech, Shanghai, China, Cat. #A610451) was added, followed by incubation at 37 °C for 5 min. The released nuclei were filtered through a 20 μm cell strainer (pluriSelect, Wolfen, Germany, Cat. #43-10020-20) and washed twice with wash buffer. A subset of nuclei was stained with DAPI (Abcam, Cambridge, UK, Cat. #ab228549), loaded onto a hemocytometer, and examined under an inverted fluorescence microscope (Nikon Eclipse Ts2-FL, Tokyo, Japan) to assess nuclear integrity. Qualified single nuclei were subsequently processed for snRNA-seq following the VITA library construction workflow. Microdroplet generation, single-nucleus encapsulation, and nucleic acid capture were performed using the VITAcruizer DP400 single-cell preparation system (M20 Genomics, Hangzhou, China, Cat. #E20000131). Library preparation and purification were completed using the VITApilote High-Throughput FFPE Single-Cell Transcriptome Kit (M20 Genomics, Hangzhou, China, Cat. #R20123124). Final libraries were sequenced on an Illumina NovaSeq 6000 platform. Single-nucleus RNA-seq analysis was conducted in Seurat (v4.1.1) with default settings unless stated otherwise.

Clustering and annotation: Following quality filtering, data were normalized and scaled using SCTransform (Seurat v4.1.1), and highly variable genes were identified. PCA was run with RunPCA, and significant principal components were used for clustering and UMAP embedding. Clusters were defined with the FindClusters function, and differentially expressed genes were identified with FindAllMarkers. Cell types were annotated using canonical marker genes.

Cell–Cell Communication Analysis: To compare ligand–receptor signaling patterns across the two biological groups (PR and CR), we used the CellChat-derived communication objects and extracted their aggregated cell–cell interaction matrices.

Analysis of Single-Cell Trajectories: The RNA assay was set as an active assay, and the raw count matrix, together with derived cell metadata from Seurat, was converted into a Monocle2 CellDataSet using a negative-binomial expression family. Lowly or noninformative genes were removed, and ordering genes were defined by intersecting the top variable features identified in Seurat with the genes retained in the Monocle2 object. Size factors and dispersions were estimated, and dimensionality reduction was performed with DDRTree (2D embedding) to learn a branched manifold. Final trajectories were visualized with Monocle2’s plotcelltrajectory function, coloring cells by group, pseudotime, or state.

### 2.5. Flow Cytometry

Tumor cells were collected and washed twice with PBS. For surface immunostaining, cells were resuspended in Cell Staining Buffer (Biolegend, San Diego, CA, USA, Cat#420201) and incubated with APC anti-CD80 antibody (Biolegend, San Diego, CA, USA, Cat#305219) for 30 min at room temperature in the dark. Cells were then washed twice in Cell Staining Buffer (5 min per wash), resuspended, and sorted by flow cytometry.

For intracellular staining, cells were fixed and permeabilized with Cyto-Fast™ Fix/Perm buffer (Biolegend, San Diego, CA, USA, Cat#426803) for 20 min at room temperature, washed twice with Cyto-Fast™ Perm Wash Solution (Biolegend, San Diego, CA, USA, Cat#426803), and incubated with primary antibodies for 20 min in the dark. After two washes, cells were incubated with the corresponding fluorophore-conjugated secondary antibody for 20 min, washed again, and finally resuspended in Cell Staining Buffer for acquisition. The primary antibodies used were anti-BMI1 antibody (Proteintech, Wuhan, China, Cat#66161-1-Ig) and anti-Caspase3 antibody (Absin Bioscience Inc., Shanghai, China, Cat#abs119676).

For sorting of ALDH^high^CD44^+^ and ALDH^low^CD44^−^ populations, cancer cells were first stained with the ALDEFLUOR assay kit (STEMCELL Technologies, Vancouver, BC, Canada, Cat#01700) following the manufacturer’s protocol and then subjected to standard surface staining with anti-CD44 antibody (BD Pharmingen™, San Diego, CA, USA, Cat#550989).

### 2.6. RT-qPCR and RNA-Seq

Total RNA was extracted using the SteadyPure Quick RNA Extraction Kit (Accurate Biotechnology, Changsha, China, Cat#AG21023) following the manufacturer’s protocol. cDNA was synthesized using a reverse transcription kit (Takara, Kyoto, Japan). Quantitative real-time PCR was carried out with SYBR Green Pro Taq HS (Accurate Biotechnology, Changsha, China, Cat#AG11736) on a QuantStudio 6 Flex Real-Time PCR System (Applied Biosystems, Foster City, MA, USA). GAPDH was used as the reference gene, and relative expression was determined by the 2^−ΔΔCt^ method. Primer sequences (Tsingke Biotech Co., Ltd., Beijing, China) are listed in Supplementary Table S2.

For RNA sequencing, total RNA was isolated with TRIzol and evaluated for integrity and purity. RNA passing quality control was used to prepare libraries with the Hieff NGS^®^ Ultima Dual-mode mRNA Library Prep Kit (Yeasen Biotechnology, Shanghai, China, Cat#12309ES), followed by sequencing on an Illumina NovaSeq X Plus platform. Raw reads were processed with fastp to remove adapters and low-quality bases, yielding clean reads for downstream analysis, which was conducted on the Omicsmart platform (http://www.omicsmart.com).

### 2.7. Tumor Sphere Formation

Tumorsphere assays were performed by plating 2 × 10^4^ SCC cells in serum-free DMEM/F-12 medium (Thermo Fisher Scientific, Waltham, MA, USA, Cat#11330-032) supplemented with 20 ng/mL human recombinant EGF (SinoBiological, Beijing, China, Cat#10605-HNAE), 20 ng/mL human recombinant bFGF (SinoBiological, Beijing, China, Cat#10014-HNAE), 1% B-27 (Thermo Fisher Scientific, Waltham, MA, USA, Cat#17504044) and N2 (Thermo Fisher Scientific, Waltham, MA, USA, Cat#17502048), in ultra-low-attachment plates (Costar, Corning, NY, USA). After 10 days of culture, spheres with a diameter of at least 70 µm were quantified.

### 2.8. Cell Proliferation Assay

Cell viability was determined using the CCK-8 assay (Dojindo Laboratories, CK40, Kumamoto, Japan) according to the manufacturer’s instructions. Briefly, 2000 cells per well were seeded into standard 96-well polystyrene plates in 100 μL of DMEM containing 10% FBS. At the indicated time points (0–96 h), CCK-8 reagent was added, and absorbance was measured using an ELx808 microplate reader (BioTek, Winooski, VT, USA).

### 2.9. Migration and Invasion Assay

For migration and invasion assays, Transwell filter inserts (Costar, Corning, NY, USA) with or without Matrigel coating (Corning, NY, USA) were placed in 24-well plates. A total of 8 × 10^4^ SCC cells were seeded into each insert. After 20 h of incubation, cells that had migrated or invaded through the membrane were fixed, stained with crystal violet, and quantified under a bright-field microscope.

### 2.10. Co-Culture and Calcein AM/PI Staining

Peripheral blood (20 mL) was collected from healthy donors, and PBMCs were isolated by Lymphoprep™ (STEMCELL, Vancouver, BC, Canada, Cat#07851). CD3^+^ T cells were purified using a MojoSort™ Human CD3 T Cell Isolation Kit (Biolegend, San Diego, CA, USA, Cat#480022) and stimulated with anti-CD3/CD28 magnetic beads (Thermo Fisher Scientific, Waltham, MA, USA, Cat#11131D), which were subsequently removed using a magnetic separator. T cells were then co-cultured with tumor cells in the presence of a CD80-blocking antibody (2 mg/mL, Santa Cruz Biotechnology, Dallas, TX, USA, Cat#sc-1177). After 48 h, tumor cells were harvested for downstream analyses. To quantify live and dead cells, cells were costained with calcein acetoxymethyl (AM) and propidium iodide (PI) (Calcein/PI Cell Viability/Cytotoxicity Assay Kit, Beyotime, Shanghai, China, Cat#C2015S) according to the manufacturer’s instructions. Briefly, the cells were washed with PBS and incubated in a PBS solution containing calcein AM and PI in the dark for 30 min at room- temperature. Then, the cells were observed and imaged using a fluorescence microscope (Olympus BX51; Olympus, Japan).

### 2.11. Syngeneic Tumor Model

All animal studies were authorized by the Institutional Animal Care and Use Committee of Peking University Health Science Center, in compliance with the Animal Ethical and Welfare Committee guidelines (approval nos. BDKQ-202501150029/approval date: 15 January 2025, BDKQ-202506100723/approval date: 10 June 2025). Healthy male C57BL/6 mice (4 weeks) were subcutaneously injected with 100 µL PBS containing 1 × 10^6^ cells. The mice were randomly divided into four groups (two groups received MOC1 cells, while the other two groups received MOC2 cells, *n* = 6 per group). Tumor size was measured using the following formula: V = length × width^2^/2. The anti-CD80 antibody (200 µg/mouse, Bio X Cell, Lebanon, NH, USA, Cat#BE0134) or IgG (200 µg/mouse, Bio X Cell, Lebanon, NH, USA, Cat#BE0089) was given via intraperitoneal injection at 2-day intervals after tumor volume reached about 50 mm^3^. Tumor volume was recorded throughout the experiment. At the conclusion of the study, all mice were anesthetized, and samples were collected for subsequent experiments.

### 2.12. Immunohistochemistry Staining

For immunohistochemistry staining, sections underwent heat-induced epitope retrieval and endogenous peroxidase quenching prior to overnight incubation with primary antibodies at 4 °C. After incubation with horseradish peroxidase-conjugated secondary antibodies, immunoreactivity was visualized using diaminobenzidine. The primary antibodies used were anti-caspase3 antibody (1:100, Absin Bioscience Inc., Shanghai, China, Cat#abs119676), anti-CD8 antibody (1:100, Santa Cruz Biotechnology, Dallas, TX, USA, Cat#sc-1177), anti-GZMB antibody (1:100, Proteintech, Wuhan, China, Cat# 13588-1-AP), anti-perforin antibody (1:100, Proteintech, Wuhan, China, Cat# 14580-1-AP), and anti-IFN-*γ* antibody (1:100, Proteintech, Wuhan, China, Cat#15365-1-AP). Images were captured using an Olympus BX51 microscope (Olympus, Tokyo, Japan).

### 2.13. Statistics

All data are presented as mean ± S.D unless otherwise specified. For comparisons between two groups, Student’s *t*-test was used, whereas one-way ANOVA was applied for comparisons among more than two groups, and two-way ANOVA was used for experiments involving multiple factors. Correlations between continuous variables were assessed using Spearman’s rank correlation test. All statistical analyses were performed with GraphPad Prism 10, and a *p* value < 0.05 was considered statistically significant.

## 3. Results

### 3.1. Single-Cell Transcriptional States of Partial Response and Complete Response to Neoadjuvant Chemoimmunotherapy in HNSCC

**Figure 1:**
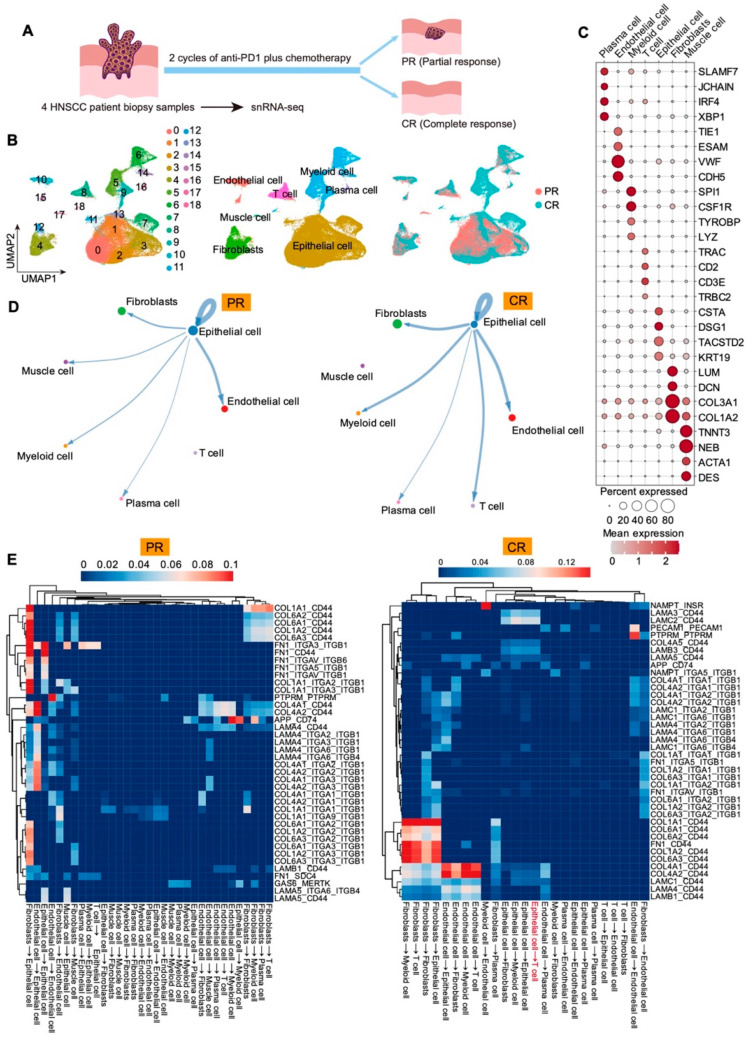
SnRNA-seq profiling of diverse cell types and cell–cell interactions in PR and CR patients. (**A**) Schematic diagrams summarize treatment regimens and group assignment (PR vs. CR) for the four patients profiled by snRNA-seq. (**B**) UMAP plots show the distribution of cell populations in four HNSCC patients, who are divided into PR and CR groups undergoing neoadjuvant therapy. PR, partial responders. CR, complete responders. *N* = 58,352. (**C**) The Dot plot shows seven cell types and the expression of the marker genes. (**D**) Cell–cell communication analysis reveals the interactions between epithelial cells and other populations in the PR and CR groups. (**E**) Heatmaps show the ligand-receptor strength between different cell populations in PR and CR.

To investigate the key determinants of neoadjuvant chemoimmunotherapy efficacy in HNSCC, we collected tumor specimens from four HNSCC patients who received two cycles of tislelizumab in combination with cisplatin and paclitaxel and subjected them to single-nucleus RNA sequencing (snRNA-seq) (Figure 1A). Clinical and pathological evaluations before and after neoadjuvant chemoimmunotherapy were used to stratify patients into partial response (PR) and complete response (CR) cohorts. CR patients achieved complete remission with no detectable residual tumor cells, whereas PR patients exhibited only partial remission with persistent tumor cells. After quality control of snRNA-seq data, 58,352 high-quality nuclei were retained and subjected to dimensionality reduction and unsupervised clustering, yielding 18 transcriptionally distinct clusters (Figure 1B). Based on canonical marker genes, these clusters were annotated with seven major cell populations: plasma cells (*SLAMF7*, *JCHAIN*, *IRF4, XBP1*), endothelial cells (*TIE1*, *ESAM*, *VWF*, *CDH5*), myeloid cells (*SPI1*, *CSF1R*, *TYROBP*, *LYZ*), T cells (*TRAC*, *CD2*, *CD3E*, *TRBC2*), epithelial cells (*KRT19*, *CSTA*, *DSG1*, *TACSTD2*), fibroblasts (*LUM*, *DCN*, *COL3A1*, *COL1A2*) and muscle cells (*TNNT3*, *NEB*, *ACTA1*, *DES*) (Figure 1C). We examined the interaction strength between epithelial cells and other populations and compared patterns between PR and CR tumors. Notably, ligand-receptor interactions between epithelial cells and T cells were virtually absent in PR tumors but pronounced in CR tumors (Figure 1D). Ligand-receptor scoring further highlighted elevated LAMC3-CD44 and COL4A5-CD44 interactions between epithelial and T cells (Figure 1E), consistent with matrix-immune adhesion and spatial anchoring that may facilitate T-cell positioning and trafficking, thereby enhancing infiltration and antitumor immunity. These data suggest that the insufficiency of epithelial-T cell crosstalk in the tumor environment may be a key mechanism driving treatment resistance.

### 3.2. A CSC Subpopulation with Enhanced Stemness Exhibits High CD80 Expression

**Figure 2:**
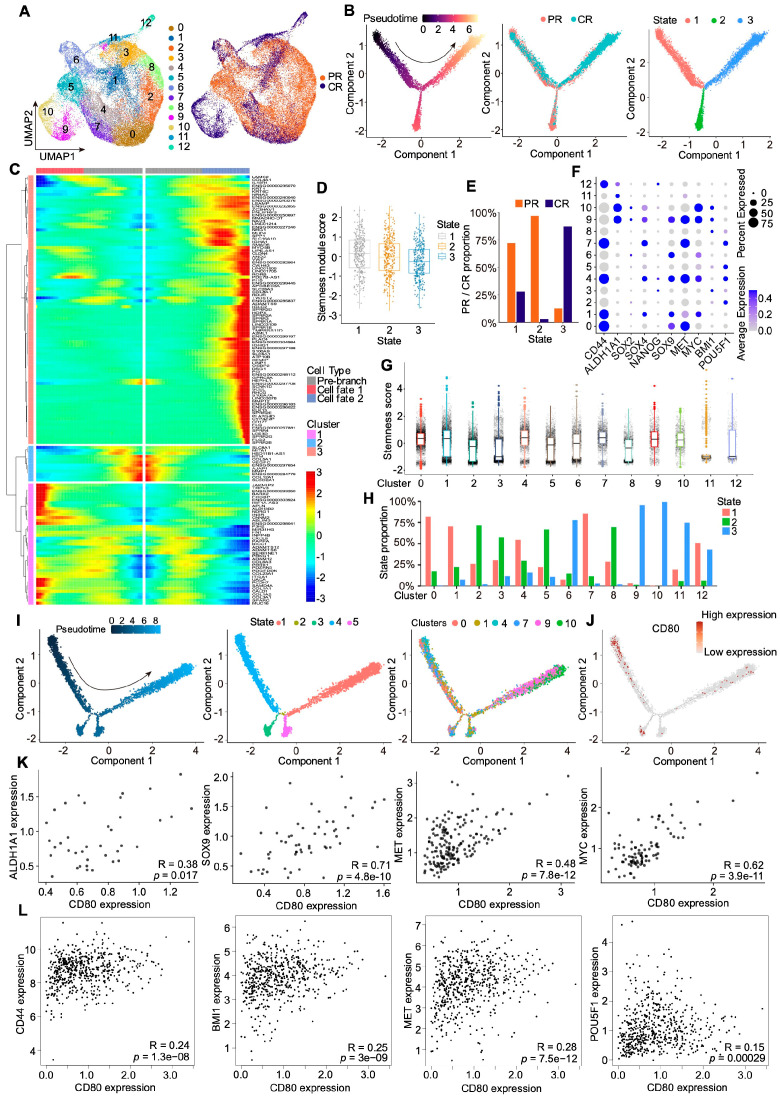
CD80 is related to cancer-cell stemness in PR and CR patients. (**A**) UMAP plots show re-clustering of epithelial cells in PR and CR groups. (**B**) Pseudotime analysis demonstrates the derivation of epithelial cells in PR and CR groups based on the derived trajectory, tissue origin, and monocle states. (**C**) The Heatmap shows the top genes expressed with the pseudotime trajectory of three fate cells. (**D**) The Boxplot shows the stemness scores of three state cells. (**E**) The PR and CR proportions in each state. (**F**) The Dot plot shows the expression levels of stemness-related genes in each cluster of epithelial. (**G**) The Boxplot shows stemness scores among different clusters of epithelial cells. (**H**) The state proportion in each cluster. (**I**) Pseudotime analysis shows the derivation of clusters 0, 1, 4, 7, 9, and 10 from epithelial cells based on monocle states and cluster origin. (**J**) Trajectory of the expression of CD80. (**K**) Analysis of the correlation between *CD80* and stemness-related genes *ALDH1A1*, *SOX9*, *MET,* and *MYC* in epithelial cells from PR and CR groups. (**L**) Analysis of the correlation between *CD80* and stemness-related genes *CD44*, *BMI1*, *MET,* and *POU5F1* in HNSCC based on TCGA HNSCC datasets.

Accumulating evidence supports the existence of a CSC subpopulation with robust self-renewal and differentiation potential in HNSCC that drives therapeutic resistance [17,20,21]. To further characterize this component, we reclustered the epithelial compartment and identified 12 discrete clusters (Figure 2A). Pseudotime analysis revealed three branches (states 1–3) in PR and CR tumors, with state 1 representing the least differentiated, most stem-like compartment (Figure 2B–D). Although state 1 contained epithelial cells from both PR and CR tumors, PR-derived cells predominated (Figure 2E). This suggests that an early, stem-like epithelial population in PR tumors may underpin resistance to neoadjuvant therapy. We then quantified stemness scores across the 12 epithelial clusters and found that clusters 0, 1, 4, 7, 9, and 10 were enriched for stemness signatures (Figure 2F,G). Notably, clusters 0, 1, 4, and 7 were largely drawn from state 1, consistent with these clusters occupying early developmental positions (Figure 2H). Trajectory inference focusing on these clusters placed 0, 1, 4, and 7 at the root of the trajectory, indicative of a primitive CSC-like state (Figure 2I). *CD80* expression was concentrated in clusters 0, 1, 4, and 7 (Figure 2J) and positively correlated with multiple stemness-associated markers within epithelial cells, including *ALDH1A1*, *SOX9*, *MET,* and *MYC* (Figure 2K). Analysis of public TCGA HNSCC data also demonstrated strong positive correlations between *CD80* and established stemness markers, such as *CD44*, *BMI1, MET*, and *POU5F1* (Figure 2L). Collectively, these findings delineate the CSC population in PR tumors characterized by high CD80 expression and suggest that *CD80* is tightly linked to treatment resistance in HNSCC.

### 3.3. CD80^+^ HNSCC Cells Possess Stronger Stemness Capacity

**Figure 3:**
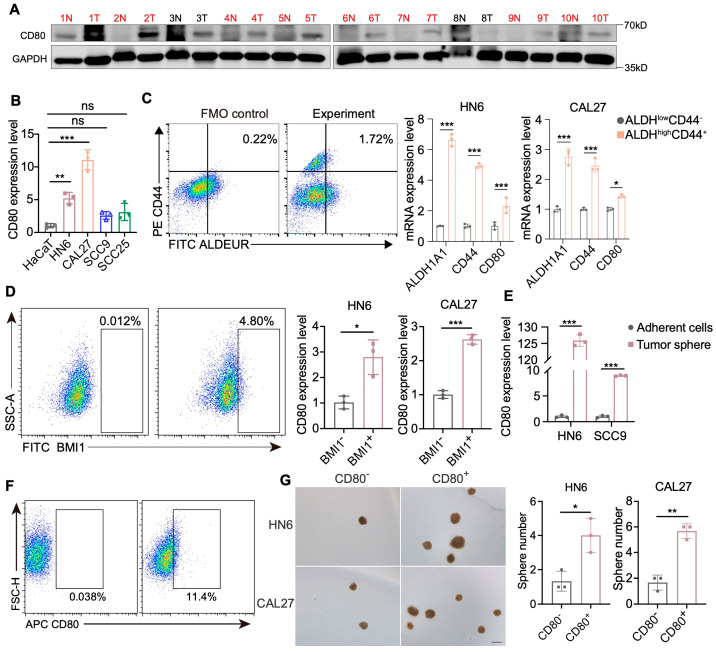
CD80 is highly expressed in CSCs. (**A**) Western blot shows protein expression levels of CD80 in 10 paired HNSCC tissues. (**B**) The expression level of CD80 in normal epithelial cell line (HaCaT) and HNSCC cell lines (HN6, CAL27, SCC9, and SCC25). Values are mean ± SD. ns, no significance; ** *p* < 0.01; *** *p* < 0.001; One-way ANOVA test; *n* = 3. (**C**) Flow cytometry isolates ALDH^high^CD44^+^ populations from HNSCC cell lines. The left panel shows the fluorescence minus one (FMO) control (the ALDH-only control). And RT-qPCR shows significantly higher CD80 expression in ALDH^high^CD44^+^ versus ALDH^low^CD44^−^ cancer cells. Values are mean ± SD. * *p* < 0.05; *** *p* < 0.001; Student’s *t* test, *n* = 3. (**D**) Flow cytometry isolates BMI1^+^ populations from HNSCC cell lines, and RT-qPCR shows significantly higher CD80 expression in BMI1^+^ versus BMI1^−^ cancer cells. Values are mean ± SD. * *p* < 0.05; *** *p* < 0.001; Student’s *t* test, *n* = 3. (**E**) RT-qPCR shows significantly higher CD80 expression in tumor sphere versus adherent cells in HNSCC cells. Values are mean ± SD. *** *p* < 0.001; Student’s *t* test, *n* = 3. (**F**) Representative images of flow-cytometry show CD80^+^ HNSCC cells. (**G**) Representative images of tumor spheres and quantification of the sphere number of CD80^−^ and CD80^+^ HNSCC cells. Scale bar, 200 μm. Values are mean ± SD. * *p* < 0.05; ** *p* < 0.01; Student’s *t* test, *n* = 3.

We next sought to validate the link between CD80 and stemness using HNSCC cell lines. CD80 was markedly upregulated in HNSCC tumor tissues compared to normal tissues (Figure 3A). Among HNSCC cell lines, HN6 and CAL27 showed high CD80 expression and were chosen for downstream functional analyses (Figure 3B). We found that CD80 expression was significantly higher in ALDH^high^CD44^+^ cells than in ALDH^low^CD44^−^ cells (Figure 3C). Consistently, CD80 was also markedly enriched in BMI1^+^ cells (Figure 3D). Moreover, CD80 expression was substantially higher in sphere-forming CSCs than in adherent counterparts (Figure 3E). These findings indicate that CD80 is preferentially expressed in CSC subpopulations and implicate CD80 in the maintenance of tumor stemness. To further test the ability of CD80^+^ cells to form tumorspheres, we sorted CD80^−^ and CD80^+^ cells by flow cytometry and seeded equal numbers of cells into tumorsphere assays. CD80^+^ cells formed significantly more tumorspheres than CD80^−^ cells (Figure 3F,G), demonstrating that CD80^+^ cells possess enhanced stemness and self-renewal potential.

### 3.4. CD80 Regulates the Stemness of CSCs and Pro-Tumorigenicity in HNSCC

**Figure 4:**
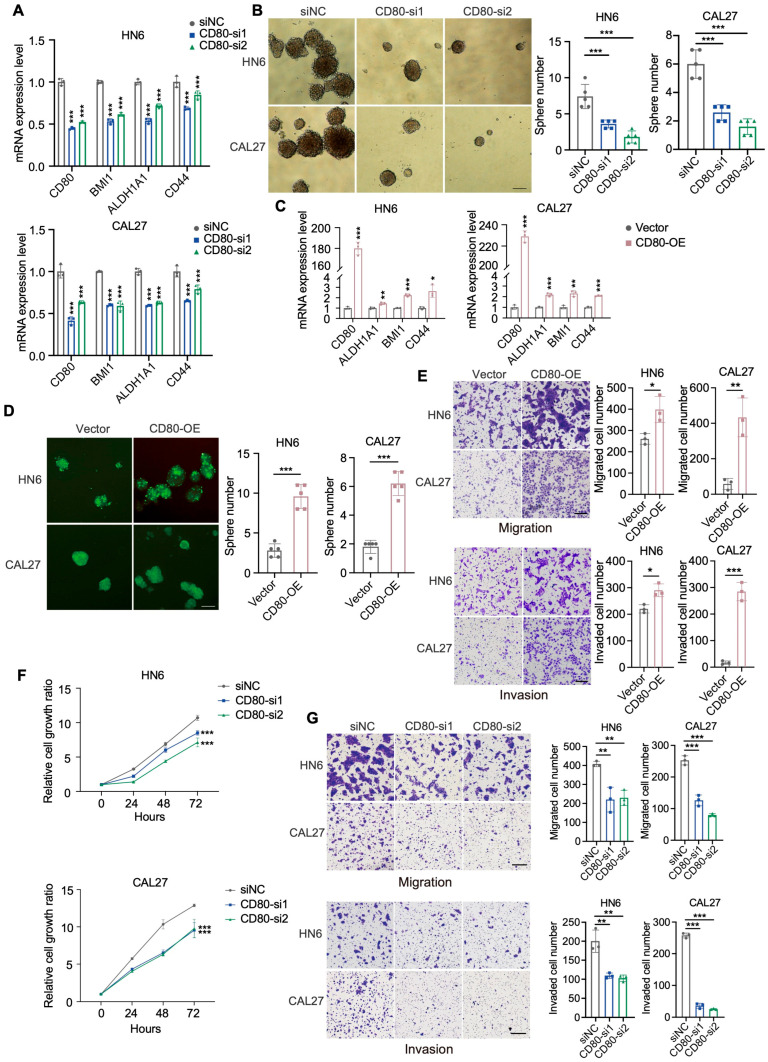
CD80 plays a crucial role in promoting cell stemness, proliferation, migration, and invasion. (**A**) RT-qPCR shows that knockdown of CD80 decreases the expression of stemness-related genes in HNSCC cells. Values are mean ± SD. *** *p* < 0.001; Two-way ANOVA test, *n* = 3. (**B**) Representative images of tumor spheres and quantification of the sphere number of HNSCC cells with knockdown of CD80. Scale bar, 200 μm. Values are mean ± SD. *** *p* < 0.001; One-way ANOVA test, *n* = 5. (**C**) RT-qPCR shows that overexpression of CD80 promotes the expression of stemness-related genes in HNSCC cells. Values are mean ± SD. * *p* < 0.05; ** *p* < 0.01; *** *p* < 0.001; Two-way ANOVA test, *n* = 3. (**D**) Representative images of tumor spheres and quantification of the sphere number of HNSCC cells with overexpression of CD80. Scale bar, 200 μm. Values are mean ± SD. *** *p* < 0.001; Student’s *t* test, *n* = 5. (**E**) Representative images and quantification of migration and invasion of HNSCC cell lines treated with CD80 overexpression. Scale bar, 100 μm. Values are mean ± SD. * *p* < 0.05; ** *p* < 0.01; *** *p* < 0.001; Student’s *t* test, *n* =3. (**F**) Knockdown of CD80 inhibits the cell proliferation of HNSCC cells. Values are mean ± SD. *** *p* < 0.001; Two-way ANOVA test, *n* = 3. (**G**) Representative images of tumor spheres and quantification of migration and invasion of HNSCC cell lines treated with CD80 knockdown. Scale bar, 100 μm. Values are mean ± SD. ** *p* < 0.01; *** *p* < 0.001; One-way ANOVA test, *n* =3.

To investigate whether CD80 can regulate tumor cell stemness, we established CD80 knockdown and overexpression models. Silencing CD80 reduced the expression of stemness-related marker genes and markedly attenuated tumorsphere formation (Figure 4A,B). In contrast, CD80 overexpression reprogrammed stemness-related transcriptional programs, leading to upregulation of stemness markers, enhanced self-renewal capacity, and increased sphere formation (Figure 4C,D). We next examined the impact of CD80 on malignant behavior. Upregulation of CD80 increased the migration and invasion of HNSCC cells, consistent with a more aggressive phenotype and potential promotion of tumor progression (Figure 4E). Conversely, CD80 downregulation weakened tumor cell proliferation and concomitantly reduced their migratory and invasive abilities (Figure 4F,G). Together, these data show that CD80 regulates both stemness and malignant properties of HNSCC cells, thereby nominating CD80 as a potential therapeutic target to suppress the pro-tumor activity of HNSCC cells.

### 3.5. CD80 Modulates the Tumor Immune Microenvironment in HNSCC

**Figure 5:**
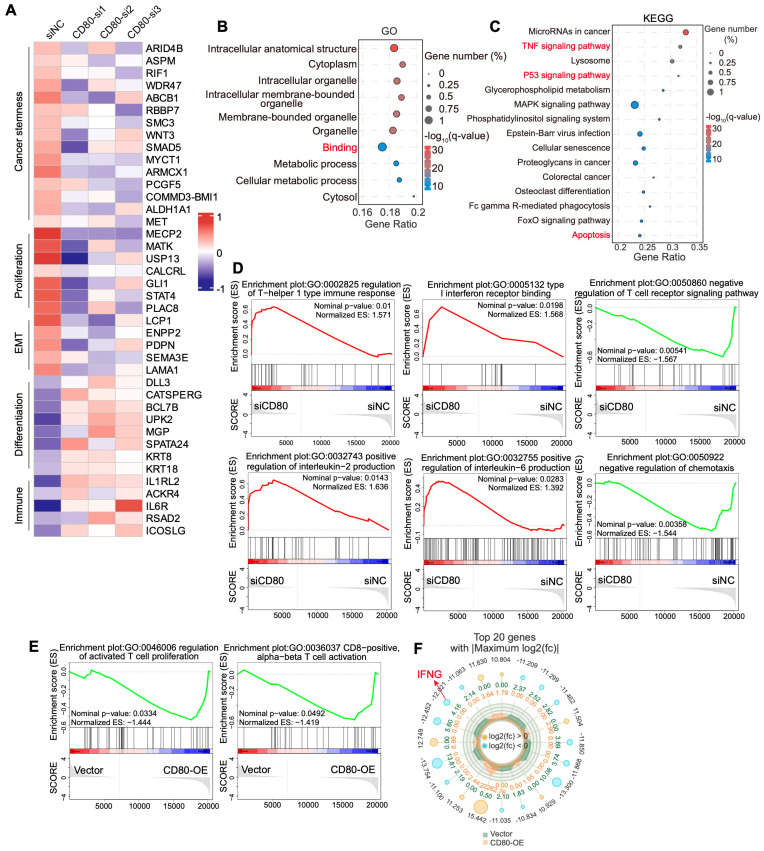
CD80 participates in antitumor immunity. (**A**) The Heatmap shows the gene expression profiles of HNSCC cells with the knockdown of CD80 by RNA-seq. (**B**) Top 10 enriched GO terms in HNSCC cells following CD80-si2 knockdown. (**C**) Top 15 enriched KEGG terms in HNSCC cells after CD80-si2 knockdown. (**D**) GSEA of the RNA-seq data from HNSCC cells with knockdown of CD80-si2. (**E**) GSEA of the RNA-seq data from HNSCC cells with overexpression of CD80. (**F**) Top 20 upregulated or downregulated genes in HNSCC cells with overexpression of CD80.

Our earlier analyses revealed pronounced differences in the interaction strength between epithelial cells and T cells in PR versus CR patients (Figure 1D), prompting us to investigate how tumor-cell-intrinsic CD80 expression regulates T-cell immunity. We therefore performed RNA-seq on HNSCC cells following CD80 knockdown and analyzed the differentially expressed genes (Figure 5A). Loss of CD80 downregulated stemness programs as well as proliferation and EMT signatures, which indicated the downregulation of malignant potential. And differentiation and immune-related genes were upregulated (Figure 5A). Gene Ontology (GO) analysis showed that CD80 knockdown significantly remodeled multiple biological processes (Figure 5B), while KEGG analysis further revealed significant alterations in TNF and P53 signaling, as well as in pathways related to autophagy and apoptosis (Figure 5C). Most of these pathways are tightly linked to tumor immunity. GSEA analysis yielded further mechanistic clues. CD80 knockdown activated a T helper 1 (Th1) immune response signature, which is known to promote CD8^+^ T-cell activation and enhance cytotoxic effector function through robust secretion of IFN-γ, TNF-α, and IL-2 (Figure 5D). Notably, type I interferon receptor-binding signaling, positive regulation of IL-2 and IL-6 production were indeed activated (Figure 5D). Moreover, pathways that suppress T-cell activation and cytokine production were downregulated (Figure 5D). In contrast, CD80 overexpression led to suppression of T-cell activation pathways, including those related to CD8^+^ αβ T-cell activation (Figure 5E). And IFNG expression was significantly downregulated (Figure 5F). Together, these data indicate that CD80 downregulation promotes T cell-mediated immune activation, whereas CD80 upregulation dampens T-cell responses.

### 3.6. Blockade of CD80 Promotes the Killing Capacity of T Cells

**Figure 6:**
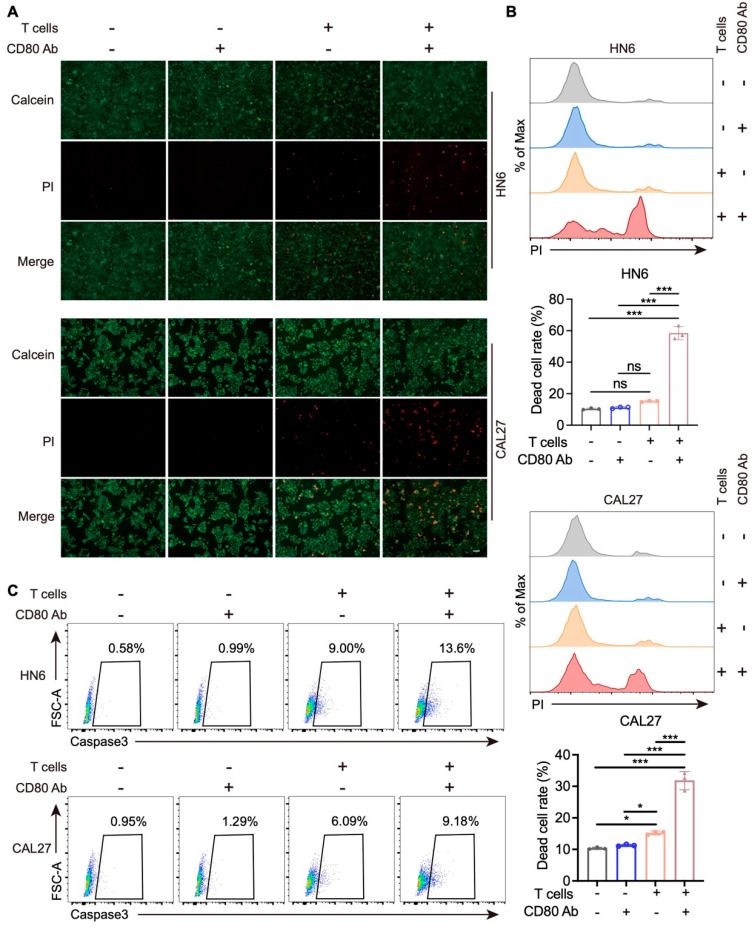
Anti-CD80 antibodies reinforce T-cell killing ability. (**A**) Calcein AM/PI staining in HNSCC cells after co-culture with or without T cells/anti-CD80 antibodies for 48 h. Scale bar, 200 μm. (**B**) Representative images and quantification of flow cytometry show the dead cell rate of HNSCC cells after co-culture with or without T cells/anti-CD80 antibodies for 48 h. Values are mean ± SD. ns, no significance; * *p* < 0.05; *** *p* < 0.001; One-way ANOVA test, *n* = 3. (**C**) Representative images of flow cytometry show the rate of caspase3^+^ HNSCC cells after co-culture with or without T cells/anti-CD80 antibodies for 48 h.

We next functionally validated the impact of tumor cell-derived CD80 on T-cell cytotoxicity. Peripheral blood mononuclear cells (PBMCs) were isolated from healthy donors, CD3^+^ T cells were purified using a T-cell isolation kit and subsequently activated with anti-CD3/CD28 antibodies. After removal of anti-CD3/CD28 beads using a magnetic separator, activated T cells were then co-cultured with HNSCC cells in the presence or absence of a CD80-blocking antibody. After 48 h, T-cell killing ability was assessed. T cells displayed only modest cytotoxic activity toward tumor cells without CD80 blockade, whereas CD80 blockade markedly enhanced their killing capacity (Figure 6A). This effect was corroborated by flow cytometric analyses, which revealed the dead cell rate significantly increased with CD80 blockade (Figure 6B). Consistently, the quantification of caspase3^+^ cells demonstrated that CD80 blockade also increased the proportion of apoptotic tumor cells (Figure 6C). Together, these findings indicate that blocking CD80 augments T cell-mediated cytotoxicity and tumor cell apoptosis, suggesting that targeting CD80 may represent a potential strategy to potentiate antitumor T-cell responses and help overcome treatment resistance.

### 3.7. CD80 Blockade Suppresses the Progression of HNSCC and Enhances Antitumor Immune Response

**Figure 7:**
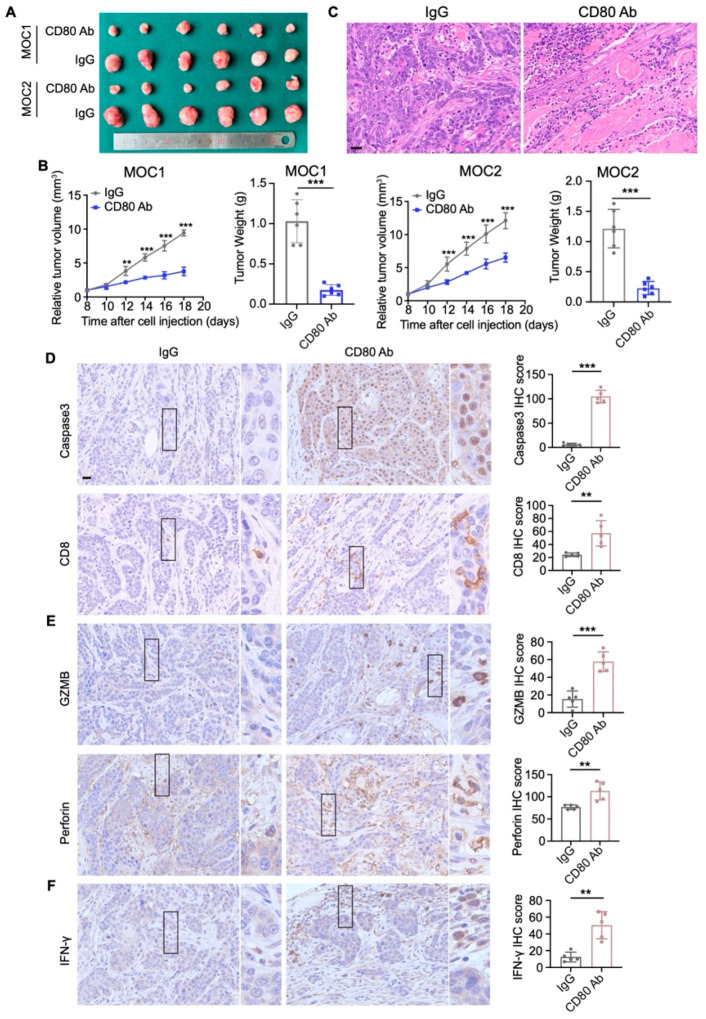
Blockade of CD80 inhibits HNSCC progression and enhances CD8^+^ T-cell function. (**A**) MOC1 and MOC2 cells were injected subcutaneously into C57BL/6 mice. Representative images show the tumors 18 days after implantation. (**B**) Tumor volume and tumor weight were significantly reduced in C57BL/6 mice treated with anti-CD80 antibody. Values are mean ± SD. ** *p* < 0.01; ***p < 0.001; Student’s *t* test; *n* = 6. (**C**) Representative H&E staining of tumors from mice injected with MOC2 cells. Scale bar, 25 μm. (**D**–**F**) Representative immunohistochemical staining and quantitative analysis of Caspase3, CD8, GZMB, Perforin, and IFN-γ from MOC2-bearing mice treated with isotype IgG or anti-CD80 antibody. Scale bar, 40 μm. Values are mean ± SD. ** *p* < 0.01; *** *p* < 0.001; Student’s *t* test; *n* = 6.

To determine whether CD80 blockade restrains HNSCC progression in vivo, MOC1 and MOC2 cells were subcutaneously implanted into C57BL/6 mice. From day 8 post-implantation, mice received intraperitoneal injections of anti-CD80 blocking antibody or isotype IgG every two days, and tumor size was monitored. From day 18 post-implantation, tumors were harvested and measured (Figure 7A). By day 12 post-implantation, tumors in the anti-CD80 group were significantly smaller than those in the control group (Figure 7B). After 10 days of treatment (day 18), tumor volumes were markedly reduced, and tumor weights were significantly lower in the anti-CD80 antibody group (Figure 7B). These findings indicate that CD80 blockade suppresses HNSCC progression. Given the higher tumorigenic potential of MOC2, which was consistent with a prior study [22], we concentrated downstream analyses on MOC2 tumors (Figure 7C). Immunohistochemistry of the tumor microenvironment revealed increased apoptosis and a significant increase in CD8^+^ T-cell infiltration following CD80 blockade (Figure 7D). Concordantly, cytotoxic effector molecules, including granzyme B (GZMB) and perforin, were elevated (Figure 7E). Moreover, IFN-γ production also increased (Figure 7F), which promoted CD8^+^ T cell-mediated antitumor immunity. Collectively, these data show that CD80 blockade inhibited HNSCC progression through enhancing CD8^+^ T-cell immunity.

## 4. Discussion

Neoadjuvant chemoimmunotherapy is promising for patients with a large tumor burden or poor status who are not suitable candidates for immediate surgery. Improving response rates to neoadjuvant chemoimmunotherapy remains a critical clinical challenge in HNSCC. We identified a CSC population that was enriched in patients who responded poorly to chemoimmunotherapy and was characterized by elevated CD80 expression. Functionally, we demonstrated that CD80 inhibition attenuates the pro-tumorigenic capacity of tumor cells and simultaneously enhances T cell-mediated cytotoxicity, thereby unveiling a promising therapeutic strategy for patients who are refractory to current neoadjuvant regimens.

It is known that tumors constitute a highly complex microenvironment composed of diverse cellular and stromal elements engaged in extensive bidirectional interactions [23,24]. We analyzed the differences in interactions between the PR and CR groups. Compared with PR patients, CR patients displayed markedly stronger interactions between epithelial cells and T cells. This is consistent with previous studies showing that crosstalk between tumor cells and T cells plays a pivotal role in tumor initiation, metastasis, and therapeutic resistance [25,26]. We also observed enhanced interactions between endothelial cells and T cells, as well as between fibroblasts and T cells in the CR group, which were proved to modulate antitumor immunity in previous studies [27,28,29]. Considering the pronounced differences in epithelial cell proportions between PR and CR tumors, we focused on the mechanisms governing epithelial cell and T cell interactions in the context of response to neoadjuvant therapy.

Trajectory inference revealed that the most primitive CSC population was predominantly derived from PR tumors, indicating that CSCs may contribute significantly to the poor response to neoadjuvant therapy. Previous studies also demonstrated the role of CSCs in treatment resistance [30]. Our analysis also defined the notably elevated CD80 expression in CSCs, which was consistent with a previous study [20]. This important feature provided a precise basis for subsequent therapeutic targeting. Moreover, we delineated a mechanistic link between CD80 and malignance, offering a concrete rationale for future CD80-targeted therapy. The absence of non-responder (NR) specimens, due to challenges in sample acquisition, limits the scope of this study. We are actively expanding the cohort, and future analyses, including NR cases, will deepen mechanistic insights into CD80 function.

We found that CD80 knockdown was accompanied by activation of T helper 1 (Th1) immune-response-related signaling pathways in tumor cells, which suggests the activation of antitumor immunity [31,32,33]. Consistently, type I interferon receptor binding and IL-2- and IL-6-related signaling pathways were positively enriched, indicating that reduced CD80 expression activates cytokine-related immune programs in tumor cells. While our study primarily characterized the phenotypic correlation between CD80 expression on CSCs and therapy resistance in HNSCC, the detailed mechanisms of CD80 action require further investigation. We attempted to explore the potential mechanisms underlying the effects of CD80 antibody blockade. CTLA-4 and CD28 are both expressed on T cells [34]. Although CD80 can bind CD28 to promote T-cell activation, its interaction with CTLA-4 instead induces immunosuppression and immune evasion. Notably, CD80 has a markedly higher binding affinity for CTLA-4 than for CD28 [35,36]. Under conditions of poor immune responsiveness, CTLA-4 expression is more likely to be increased, thereby favoring CD80–CTLA-4 engagement and suppressing T-cell activity [37]. The anti-CD80 antibody galiximab modulates immune responses by blocking the CD80–CTLA-4 pathway, thereby enhancing therapeutic efficacy in follicular lymphoma and Hodgkin lymphoma [38,39]. In this study, we found that CD80 was highly expressed in CSCs from patients with poor responses to neoadjuvant therapy. In this context, CD80 expressed on CSCs is likely to mediate immune evasion through preferential binding to CTLA-4. Furthermore, CD80 can bind with PD-L1 both by trans-interaction and cis-interaction [35,40,41]. Trans-interaction between CD80 and PD-L1 can suppress T-cell function [40,41]. In contrast, soluble CD80-Fc blocks the interaction between CD80 and PD-L1 and promotes T-cell activation. Soluble CD80-Fc also competitively occupies the binding interfaces of PD-1–PD-L1 and CD80–CTLA-4, which effectively alleviates immune evasion [42,43]. Therefore, the anti-CD80 treatment used in our co-culture assays might both block the CD80/CTLA-4 inhibitory axis and the CD80/PD-L1 pathway, thereby restoring T-cell anti-tumor efficacy. Notably, although CD86 and CD80 are both members of the B7 family and share high structural similarity [44], our preliminary analyses indicated that CD86 was not highly expressed in CSCs. Nevertheless, further studies are required to elucidate the precise mechanisms by which CD80 blockade restores T-cell immunity.

Regarding future therapeutic options for HNSCC, monoclonal antibodies (mAbs) targeting CD80, such as Galiximab, might offer an effective approach to disrupt the CSC-mediated immune evasion [45]. Compared to CAR-T and CAR-NK cell therapies, which involve complex genetic engineering and potential systemic toxicities like cytokine release syndrome (CRS) [46,47,48,49], mAbs provide a more manageable safety profile. However, while mAbs rely on the patient’s existing immune repertoire, CAR-T/NK therapies offer superior precision by directly engineering immune cells to recognize specific CSC antigens [50], potentially overcoming the HLA-restricted limitations of conventional T-cell responses.

## 5. Conclusions

Our findings suggest that CD80 is enriched in CSCs and promotes the malignant behavior of HNSCC cells. CD80 is identified as a promising therapeutic target with the potential to improve responses to neoadjuvant chemoimmunotherapy in HNSCC.

## Data Availability

The original contributions presented in this study are included in the article. Further inquiries can be directed to the corresponding authors.

## Supplementary Materials

The following supporting information can be downloaded at: https://www.mdpi.com/article/10.3390/cells15030266/s1, Figure S1: An overview of the case selection process; Table S1: Clinical characteristics of HNSCC patients for single-nucleus RNA-sequencing; Table S2: Primers used in this study; Table S3: Antibodies used in the study; Table S4: Clinical characteristics of HNSCC patients for western blot.

## Abbreviations

HNSCC, head and neck squamous cell carcinoma; CSC, cancer stem-like cell; PR, partial response; CR, complete response; PD-1, programmed death 1; Th1, T helper 1 cell; IL-2, interleukin-2; snRNA-seq, single-nucleus RNA sequencing; LAMC3, laminin subunit gamma 3; EMT, epithelial-mesenchymal transition; GO, gene ontology; KEGG, kyoto encyclopedia of genes and genomes; TNF, tumor necrosis factor; PBMCs, peripheral blood mononuclear cells.
