# Supplementary material for: CD80-Mediated T-Cell Suppression by Cancer Stem-like Cells in Head and Neck Squamous Cell Carcinoma
Source: Cells. 2026 Jan 30;15(3):266. doi: 10.3390/cells15030266 (PMC12896438; doi:10.3390/cells15030266)
Supplement: Supplementary file 1 [file cells-15-00266-s001.zip › Supplemental Table S1.pdf]

Supplemental Table S1. Clinical characteristics of HNSCC patients for single-nucleus RNA-sequencing.

Patient	Age	Sex	Histology	Tumor location	Pre-treatment clinical stage	Post-treatment pathological stage	Response evaluation[#]	snRNA-seq
P001	50	Male	HNSCC	Tongue	III (T4N2M0)	ypT2N2M0	PR	Biopsy tumor
P002	77	Female	HNSCC	Buccal mucosa	III (T3N2M0)	ypT2N2M0	PR	Biopsy tumor
P003	68	Male	HNSCC	Oropharynx	III (T3N2M0)	ypT0N0M0	CR	Biopsy tumor
P004	51	Male	HNSCC	Oropharynx	IV (T3N3M0)	ypT0N0M0	CR	Biopsy tumor

[#] CR (complete response, ypT0N0M0) was defined by the absence of residual viable cancer cells in the primary HNSCC tumor and lymph node surgical specimens, and PR (partial response, >ypT0/>ypN0) was defined as persistence or any pathologically detected residual cancer cells.
