# Supplementary material for: CD80-Mediated T-Cell Suppression by Cancer Stem-like Cells in Head and Neck Squamous Cell Carcinoma
Source: Cells. 2026 Jan 30;15(3):266. doi: 10.3390/cells15030266 (PMC12896438; doi:10.3390/cells15030266)
Supplement: Supplementary file 1 [file cells-15-00266-s001.zip › Supplementary Figure S1.pdf]

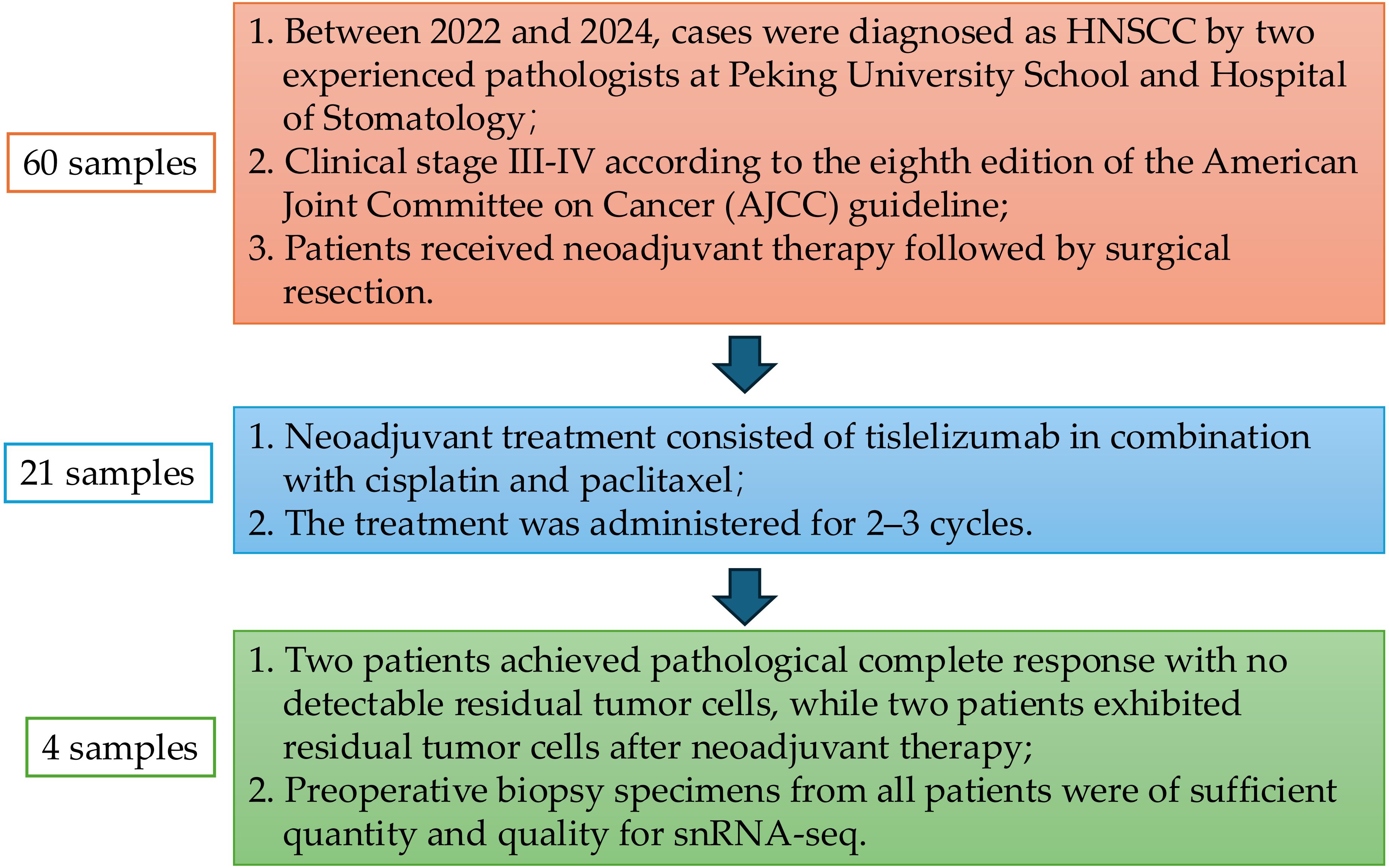
60 samples
1. Between 2022 and 2024, cases were diagnosed as HNSCC by two experienced pathologists at Peking University School and Hospital of Stomatology;
2. Clinical stage III-IV according to the eighth edition of the American Joint Committee on Cancer (AJCC) guideline;
3. Patients received neoadjuvant therapy followed by surgical resection.
21 samples
1. Neoadjuvant treatment consisted of tislelizumab in combination with cisplatin and paclitaxel;
2. The treatment was administered for 2–3 cycles.
4 samples
1. Two patients achieved pathological complete response with no detectable residual tumor cells, while two patients exhibited residual tumor cells after neoadjuvant therapy;
2. Preoperative biopsy specimens from all patients were of sufficient quantity and quality for snRNA-seq.
