# Supplementary material for: CD80-Mediated T-Cell Suppression by Cancer Stem-like Cells in Head and Neck Squamous Cell Carcinoma
Source: Cells. 2026 Jan 30;15(3):266. doi: 10.3390/cells15030266 (PMC12896438; doi:10.3390/cells15030266)
Supplement: Supplementary file 1 [file cells-15-00266-s001.zip › Supplementary Table S2.pdf]

Supplementary Table S2. Primers used in this study

Gene	Assay	Forward (5′-3′)	Reverse (5′-3′)
GAPDH	qRT-PCR	GAGTCCACTGGCGTCTTCAC	TTCACACCCATGACGAACAT
CD80	qRT-PCR	CTCACTTCTGTTCAGGTGTTA TCCA	TCCTTTTGCCAGTAGATGCGA
BMI1	qRT-PCR	TGCTTTGGTCGAACTTGGTG	TTTGCAGACTGGGGACAATG
ALDH1A 1	qRT-PCR	CGGGAAAAGCAATCTGAAGA GGG	GATGCGGCTATACAACACTGGC
CD44	qRT-PCR	CTGCCGCTTTGCAGGTGTA	CATTGTGGGCAAGGTGCTATT
CD80 siRNA1	Knockdown	CATCAAGTATGGACATTTA	
CD80 siRNA2	Knockdown	GCAGCAAACTGGATTTCAA	
CD80 siRNA3	Knockdown	GTCACAATGTTTCTGTTGA	
