# Supplementary material for: CD80-Mediated T-Cell Suppression by Cancer Stem-like Cells in Head and Neck Squamous Cell Carcinoma
Source: Cells. 2026 Jan 30;15(3):266. doi: 10.3390/cells15030266 (PMC12896438; doi:10.3390/cells15030266)
Supplement: Supplementary file 1 [file cells-15-00266-s001.zip › Supplementary Table S3.pdf]

Supplemental Table S3. Antibodies used in the study.

Antibodies	SOURCE	IDENTIFIER
PE anti-human CD44 antibody	BD Pharmingen™	Cat#550989; RRID: AB_394000
Mouse monoclonal anti-BMI1 antibody	Proteintech	Cat#66161-1-Ig; RRID: AB_2881557
APC anti-human CD80 antibody	Biolegend	Cat#305219; RRID: AB_2291403
Rabbit polyclonal anti-Caspase 3 antibody	Absin Bioscience Inc.	Cat#abs119676
InVivoMAb anti-mouse CD80	Bioxcell	Cat#BE0134; RRID: AB_10950113
InVivoMAb anti-mouse IgG	Bioxcell	Cat#BE0089; RRID: AB_1107769
Mouse monoclonal anti-CD8 antibody	Santa Cruz Biotechnology	Cat#sc-1177; RRID: AB_627185
Rabbit polyclonal anti-Granzyme B antibody	Proteintech	Cat#13588-1-AP; RRID: AB_2114429
Rabbit polyclonal anti-Perforin antibody	Proteintech	Cat#14580-1-AP; RRID: AB_10639524
Rabbit polyclonal anti-IFN-gamma antibody	Proteintech	Cat#15365-1-AP; RRID: AB_2123037
Mouse monoclonal anti-GAPDH antibody	Proteintech	Cat#60004-1-Ig; RRID: AB_2107436
Alexa Fluor 488-labeled goat anti-rabbit IgG (H+L)	Beyotime	Cat#A0423; RRID: AB_2891323
Alexa Fluor 488-labeled goat anti-mouse IgG (H+L)	Beyotime	Cat#A0423; RRID: AB_2891323
