# Supplementary material for: CD80-Mediated T-Cell Suppression by Cancer Stem-like Cells in Head and Neck Squamous Cell Carcinoma
Source: Cells. 2026 Jan 30;15(3):266. doi: 10.3390/cells15030266 (PMC12896438; doi:10.3390/cells15030266)
Supplement: Supplementary file 1 [file cells-15-00266-s001.zip › Supplementary Table S4.pdf]

Supplemental Table S4. Clinical characteristics of HNSCC patients for western blot.

Patient	Age	Sex	Histology	Tumor location	Clinical stage	TMN
P1	49	Male	HNSCC	Tongue	Ⅲ	T2N1M0
P2	54	Female	HNSCC	Tongue	Ⅲ	T2N1M0
P3	81	Female	HNSCC	Tongue	Ⅱ	T1N0M0
P4	81	Male	HNSCC	Oropharynx	Ⅱ	T2N0M0
P5	44	Female	HNSCC	Tongue	Ⅲ	T2N1M0
P6	61	Male	HNSCC	Tongue	Ⅱ	T4N2M0
P7	71	Female	HNSCC	Tongue	Ⅱ	T2N0M0
P8	48	Male	HNSCC	Tongue	Ⅳ	T4N2M0
P9	59	Male	HNSCC	Tongue	Ⅳ	T3N2M0
P10	38	Female	HNSCC	Tongue	Ⅱ	T2N0M0
